# Could *Japonica* Rice Be an Alternative Variety for Increased Global Food Security and Climate Change Mitigation?

**DOI:** 10.3390/foods10081869

**Published:** 2021-08-12

**Authors:** Daniel Dooyum Uyeh, Senorpe Asem-Hiablie, Tusan Park, Kyungmin Kim, Alexey Mikhaylov, Seungmin Woo, Yushin Ha

**Affiliations:** 1 Department of Bio-Industrial Machinery Engineering, Kyungpook National University, Daegu 41566, Korea; uyehdooyum@gmail.com (D.D.U.); woosm7571@gmail.com (S.W.); 2 Upland-Field Machinery Research Centre, Kyungpook National University, Daegu 41566, Korea; 3 Smart Agriculture Innovation Center, Kyungpook National University, Daegu 41566, Korea; 4 Institutes of Energy and the Environment, The Pennsylvania State University, University Park, PA 16802, USA; senorpe.ah@gmail.com; 5 Division of Plant Biosciences, School of Applied Biosciences, College of Agriculture & Life Science, Kyungpook National University, Daegu 41566, Korea; kkm@knu.ac.kr; 6 Financial Research Institute of Ministry of Finance of the Russian Federation, 127006 Moscow, Russia; Alexeyfa@ya.ru

**Keywords:** rice cultivation, rice carbon emissions, hidden hunger, parboiling, rice quality, rice preference

## Abstract

The growing importance of rice globally over the past three decades is evident in its strategic place in many countries’ food security planning policies. Still, its cultivation emits substantial greenhouse gases (GHGs). The *Indica* and *Japonica* sub-species of *Oryza sativa* L. are mainly grown, *with Indica* holding the largest market share. The awareness, economics, and acceptability of *Japonica* rice in a food-insecure *Indica* rice-consuming population were surveyed. The impact of parboiling on *Japonica* rice was studied and the factors which most impacted stickiness were investigated through sensory and statistical analyses. A comparison of the growing climate and greenhouse gas emissions of *Japonica* and *Indica* rice was carried out by reviewing previous studies. Survey results indicated that non-adhesiveness and pleasant aroma were the most preferred properties. Parboiling treatment altered *Japonica* rice’s physical and chemical properties, introducing gelatinization of starch and reducing adhesiveness while retaining micronutrient concentrations. Regions with high food insecurity and high consumption of *Indica* rice were found to have suitable climatic conditions for growing *Japonica* rice. Adopting the higher-yielding, nutritious *Japonica* rice whose cultivation emits less GHG in these regions could help strengthen food security while reducing GHGs in global rice cultivation.

## 1. Introduction

Recent World Bank reports show that half of the world’s poorest people are in just five countries on two continents [1]. These are India and Bangladesh in South Asia, and Nigeria, the Democratic Republic of Congo, and Ethiopia in Sub-Saharan Africa. Rice is a highly consumed staple in three of these countries with reported values as follows: Bangladesh (260 kg/capita and 36.1 Megatonnes); India (103 kg/capita and 104 Megatonnes), Nigeria (46.3 kg/capita 6.60 Megatonnes), Democratic Republic of Congo (29.7 kg/capita and 0.47 Megatonnes), and Ethiopia (4.69 kg/capita and 0.66 Megatonnes) [1,2]. Two of these countries are also among the ten top global importers of rice, with Nigeria at 1.8 Megatonnes spending USD 8.5 billion and Bangladesh at 0.9 Megatonnes spending USD 4.03 billion [3]. In these countries, there are high rates of malnourishment and food insecurity [4].

Food security has been defined in various ways. As reported by the Food and Agriculture Organization (FAO) of the United Nations, the initial focus was on the volume and stability of food supplies. This definition has evolved into “when all people always have physical, social and economic access to enough, safe and nutritious food which meets their dietary needs and food preferences for an active and healthy life” (FAO, 2019) [5]. According to this latest definition, 820 million food-insecure people [6] live in poor countries. However, “hidden hunger” becomes pertinent due to challenges such as inadequate intake of calories to support an individual’s daily activities, insufficient protein to support growth, and deficiencies in mineral and vitamins intake [7]. Serious health outcomes of hidden hunger have been recorded in most developing countries [8,9] and have been predicted to grow exponentially in Africa at a rate of 18% annually [10].

Rice, a monocot cereal customarily grown as an annual plant, is an excellent source of complex carbohydrates. As a staple for more than half of the world’s population, worldwide production has been growing steadily [11] in response to increasing demands over the past three decades. The growing importance of rice globally is evident in the strategic food security planning policies of many countries. While, currently, nine out of every ten people in the world who consume rice are Asian, rice is ranked as the fastest growing staple in Africa. In Latin America and the Caribbean, it is gaining increased acceptance [12].

By 2039, the total land area of rice harvest is projected to increase by 11% to meet the world’s needs [13]. However, rice cultivation is also a major source of greenhouse gas (GHG) emissions, with annual levels in 2017 recorded at 924 Megatonnes of carbon dioxide equivalents (CO_2_e), ahead of hard coal mining (790 Megatonnes CO_2_e) [14]. The total land area used to cultivate cereals globally is nearly 7.31 × 10^8^ ha [15]. Rice harvests account for about 23.5% of the total harvested cereal area and 27% of global cereal production and consumption [16].

Rice is cultivated in more than 100 countries [17]. Still, except for a few countries that have attained self-sufficiency, demand currently exceeds production in many, and large quantities of rice are imported to meet needs. As many as 21 of the 39 rice-producing countries in Africa import between 50 and 99% of their rice needs at substantial economic costs [18,19], making it unaffordable for the populace living in poverty. Furthermore, supply chain disruptions caused by occurrences such as conflicts and pandemics drive calls for securing local stores of domestic production of food staples. For example, the World Food Program [20] has warned of risks of widespread famines due to the COVID-19 global disease outbreak caused by the severe acute respiratory syndrome coronavirus 2 (SARS-CoV-2).

Nigeria, Africa’s second most populous country, was reported to be the second largest rice importer in the world [21] and in terms of calories consumed, rice ranked fourth. Although Nigeria was reported as the largest rice producer in West Africa, productivity is relatively low. The major constraint to growing rice in Africa is reliable water availability, as unsteady and low rainfall patterns results in crop vulnerability to drought.

Only two rice species (the African *Oryza glaberrima* and the Asian *Oryza sativa*) are reportedly cultivated in the world [22]. These are morphologically similar but differ ecologically. *O. glaberrima* has the important characteristics of being more disease and pest resistant compared to the Asian *O*. *sativa* [22]. Additionally, *O. glaberrima* is less susceptible to weeds given its broader leaves that shade weeds out. Furthermore, the African variety has a better tolerance for fluctuations in water depth, severe climates, iron toxicity, and infertile soils. A faster maturity rate gives *O. glaberrima* high potential as an important emergency food [23]. The higher yields of the Asian *O. sativa*, however, present an advantage over *O. glaberrima* whose grains are brittle and more challenging to mill. Hybrids of the two main species have been produced to preserve the desirable traits in both. Cultivation of *O. glaberrima* is predominantly in Sub-Saharan Africa but it is being replaced by *O. sativa.*

The Asian rice species, *Oryza sativa*, is the primary type cultivated worldwide and its subspecies, *Indica* and *Japonica*, are the most grown. *Indica* rice, also known as long grain, grows well near the equator with a kernel four to five times longer than its width and it holds over 80% of the global rice market share [24].

Rice quality is primarily assessed based on physical properties such as head rice yield, chalkiness, grain size and shape, grain color, adhesiveness, protein and amylose content, and aroma [25,26,27]. These properties of rice affect its physicochemical and cooking properties and, consequently, desirability among consumers. Amylose content and alkali digestion are widely recognized as important determinants of consumption quality because they reflect the functionality of the rice grain’s starch [28]. Protein content also plays an essential role in determining quality, as does the grain texture and the surface hardness of cooked rice [29].

The parboiling process of harvested paddy improves quality by producing mostly desirable biochemical, physical, and sensory changes according to reports by Oli et al. [30]; Mir and Bosco [31]; Bello et al. [32] and da Fonseca et al. [33]. The gelatinization of starch in the rice occurs during parboiling, resulting in loss of adhesiveness and producing a desirable aroma. Parboiling involves partially boiling the paddy in its husk and consists of three basic steps: soaking, steaming, and drying, as Miah et al. [34] described. During the parboiling process, the first stage of soaking rice in warm water allows the water-soluble nutrients to penetrate the endosperm from the husk. The husk contains 0.05–0.07 mg of riboflavin, 0.09–0.21 mg thiamin, and 1.6–4.2 mg niacin [35,36]. Riboflavin is slightly soluble in water, thiamin is highly soluble, and niacin is poorly soluble in water.

During the cooking process, starch granules in the grain expand, and amylose leaches into the boiling water to form a viscous solution. As the cooked rice cools, the leached amylose chains line up, lock together, and form a gel, resulting in adhesiveness. In theory, the higher the amylose content of rice, the firmer the cooked grain of rice would be. Amylose is also responsible for the way that rice hardens upon cooling. When rice cools to room temperature or lower, retrogradation occurs as the amylose chains and linear parts of amylopectin crystallize by forming hydrogen bridges.

The *Indica* paddy is usually treated to a parboiling process, resulting in the milled grains being fluffy with separate kernels when cooked. The rice seeds are also processed into other products, including pasta, rice cakes, flour, and animal feed. *Japonica* rice, commonly referred to as medium and short grain, has kernels two to three times longer than its width and grows mostly in temperate regions. It is commonly found in the Republic of Korea, Japan, the Democratic People’s Republic of Korea, and parts of China, Australia, and the United States of America. The *Japonica* rice paddy is usually not treated with any process, and the milled grains are moist, adhesive, and bright white in color. The glutinous (adhesiveness) nature of *Japonica* makes it most ideal for Mediterranean and Asian dishes that require adhesiveness, such as paella, risotto, and sushi.

The objectives of this study were to: (1) determine the awareness, acceptability, and economics of *Japonica* rice using structured questionnaires in a major parboiled *Indica* rice-consuming market; (2) analyze the differences in nutritional and physical properties of untreated *Japonica*, treated *Japonica*, and treated *Indica rice*; (3) compare the growing climate and greenhouse gas emissions of *Japonica* and *Indica* rice using previous studies; and discuss the implications for food security in food-insecure *Indica* rice-consuming markets.

## 2. Materials and Methods

A survey of consumer rice preference was performed, and analyses of the impact of parboiling on the quality of *Japonica* rice considering consumer preference were carried out. Following Adhikari et al. [37], moisture, temperature, adhesion, and quantity of low molecular sugars were measured to assess adhesiveness. Furthermore, color and nutrient levels in the rice samples were measured using spectrophotometric and wet chemistry methods.

### 2.1. Survey of Consumer Rice Preferences Using a Structured Questionnaire

A survey was administered to 1057 literate and non-literate randomly selected participants in Nigeria’s six main geographical regions (North-east, North-west, North-central, South-east, South-west, and South-south). A total of 506 literate respondents participated via an online questionnaire and in-person interviews were administered to the 551 non-literate respondents. To determine the participants’ rice preferences when presented with choices, the information gathered through the survey (Appendix B) included the following:

- knowledge of the rice sub-species and preference of sub-species,
- length of grain, adhesiveness, aroma, and taste after cooking as well as the cooking time,
- willingness to purchase healthier rice at a relatively higher cost (USD 1 = NGN 410).

The survey results were summarized using descriptive statistics.

### 2.2. Analyses of the Impact of Parboiling on the Properties of Japonica Rice

#### 2.2.1. Rice Parboiling Procedure

Samples for this study were collected from a *Japonica* paddy harvested in Dalseong gun (Republic of Korea). The rice samples were parboiled using hydrothermal and mechanical treatments as described below.

Nine *Japonica* rice samples weighing 200 g each were soaked in a water bath shaker as described in [38]. Fabricated parboiling equipment [38] was used to treat the samples under the different conditions, given in Table 1. The equipment consisted of a steaming chamber with a perforated aluminum sample holder, a digital control system, a relay switch, pressure gauge, pressure valve, safety valve, and water inlet and outlet points. Heat loss was reduced by insulating the parboiling device with a 10 mm layer of polyurethane foam. The digital control system regulated steam temperature. The pressure was controlled with an installed pressure gauge and safety valves.

Following the hydrothermal treatments, 100 g each of the nine rice samples were dried, and their moisture contents determined. Drying was carried out using a hot air oven (VS-120203N, Vision Scientific, Daejeon, Korea) [39] which was also used in moisture content determination [40], using Equations (1)–(3).

(1) $$MC_{wb}=\frac{MC_{i}-MC_{f}}{MC_{i}}\times 100$$

(2) $$MC_{db}=\frac{MC_{i}-MC_{f}}{MC_{f}}\times 100$$

Weight loss during drying:

(3) $$MC_{f}=MC_{i}\times \frac{100\times MC_{wb}}{100-MC_{wb}}$$

where $MC_{wb}$ is moisture content on a wet basis, $MC_{db}$ is moisture content on a dry basis, $MC_{i}$ is initial weight, and $MC_{f}$ are final weight.

The dried *Japonica* rice samples were milled following the method reported in [39]. Milling equipment included a huller (Model SY-88-TH, Ssang Yong, Yongin-si, Korea), abrasive type rice mill (Model TM05, Satake, Hiroshima-ken, Japan), friction type mill (Model SY-92-TR, Ssang Yong, Yongin-si, Korea), and large and small broke separator (Model SY2000-HRG, Ssang Yong, Yongin-si, Korea). The rice yield after milling was calculated as:

(4) $$Y_{milling}=\frac{W_{m.rice}}{W_{p.rice}}\times 100$$

where *Y_milling_* is milled rice yield, $W_{m.rice}$ is milled rice weight, and $W_{p.rice}$ is the initial weight of *Japonica* paddy.

**Figure 1:**
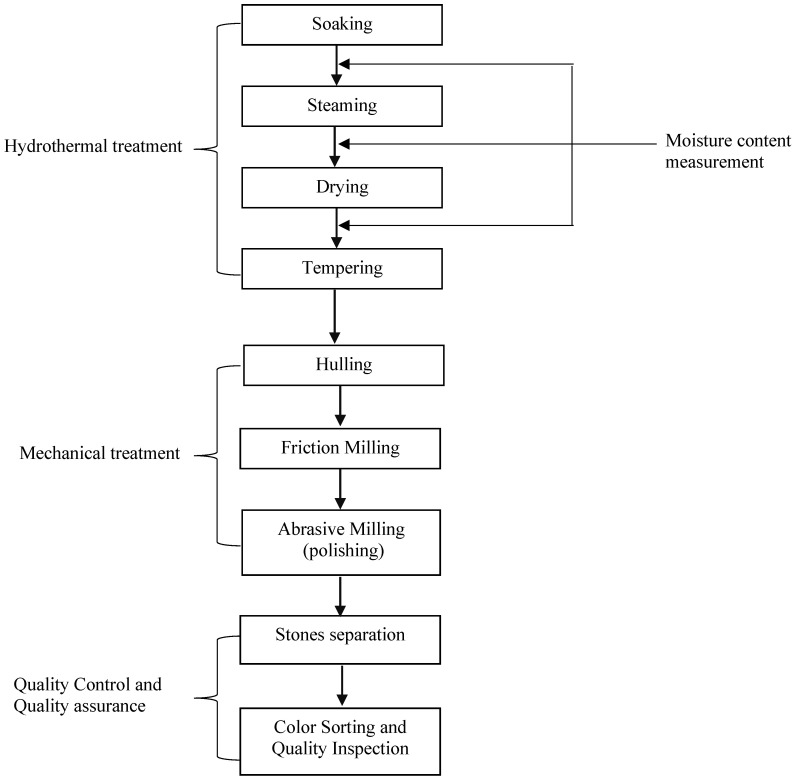
Process flow for parboiling and milling *Japonica* rice.

Sorting out of foreign materials and other quality control and quality assurance measures followed the mechanical and hydrothermal treatments. The pre-treatment processes undergone by the rice samples are shown in Figure 1.

During the parboiling treatment, the milled rice yield of *Japonica* rice was tested following an adaptation of the Taguchi method as previously described by Uyeh et al. [38], using three different settings each for moisture, steaming temperature, drying temperature, and duration of treatment (Table 1).

#### 2.2.2. Analysis of the Differences in Nutritional and Physical Properties of Milled Rice Samples

The differences in nutritional and physical properties of untreated *Japonica*, treated *Japonica*, and treated *Indica rice* were measured and analyzed. The properties measured were adhesiveness, amylose content, alkali digestion, nutrients, and color.

*a*.  *Analysis of adhesiveness in milled parboiled and non-parboiled rice samples*

**Figure 2:**
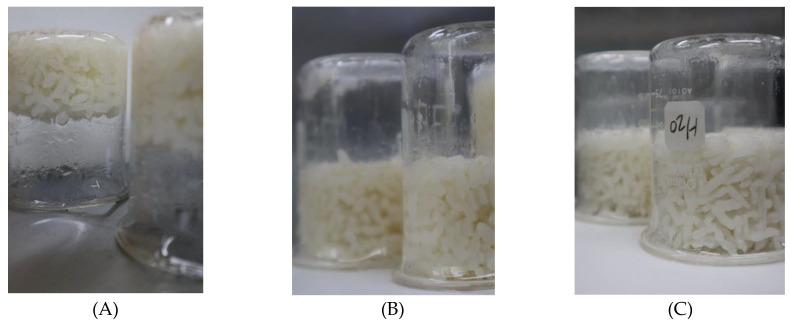
Beakers of cooked rice samples placed in inverted positions to investigate adhesion; (**A**) non-parboiled *Japonica* rice; (**B**) parboiled *Japonica* rice; and (**C**) *Indica* rice.

To measure the adhesiveness of *Japonica* and *Indica* rice, 25 g of milled triplicate samples of parboiled and non-parboiled *Japonica* and parboiled *Indica* were transferred into 100 mL beakers and placed in a home type rice cooker (DWX-220K, Morningcom, Seoul, Korea). A 40 mL volume of water was immediately transferred into each beaker of the milled rice samples, and the mixtures were stirred to a uniform consistency and evenly spread. The three beakers of samples were then placed into a rice cooker, and 400 g of water was transferred into the cooker’s pot. A thermometer was inserted into the pot to measure the cooking temperature. Twenty minutes after the temperature reached 100 °C or water vapor formed, the rice cooker was turned off and the set-up was allowed to stand for another 10 min. At this point, the rice was considered “cooked” for human consumption. The beakers were then left to stand upside-down (Figure 2) on a flat, straight, and stable surface for one hour [41]. The rice samples that stuck to the beaker were considered to be sticky.

*b*.  *Analysis of amylose content in milled parboiled and non-parboiled rice samples*

A component analyzer (AN-820, KETT, Tokyo, Japan) was used to determine the amylose content of the milled parboiled *Japonica* and *Indica* rice and raw *Japonica* rice (control) samples. The analyzer was set to the “short-milled rice” option pre-calibrated by the manufacturers before measurement.

*c*.  *Analysis of alkali digestion in milled parboiled and non-parboiled rice samples*

Alkali digestion values of milled rice samples were measured. Twenty-five-gram triplicates of each type of milled rice (*Japonica* and *Indica* rice) were placed in Petri dishes (90 × 15 mm diameter) containing 20 mL of 1.4% KOH solution. The grains were allowed to stand at 30 °C for 18 h following Choe and Heu [42]. Alkali digestion was graded from 1 to 7 referencing the seven-point scale of the International Rice Research Institute [43], as shown in Table 2 below.

*d*.  *Nutritional analyses of milled parboiled and non-parboiled rice samples*

Macronutrient (crude protein, crude fat, crude ash, and carbohydrates) and micronutrient (iron, magnesium, and zinc) contents of the parboiled *Japonica* rice and *Indica* rice and untreated *Japonica* rice were measured on a wet basis using standard methodology as described by the AOAC [44].

*e*.  *Color measurement of milled parboiled and non-parboiled rice samples*

The CIE color scales L*a*b* of cooked and uncooked for *Japonica* (parboiled and non-parboiled) and rice samples were measured with a reflectance colorimeter (CS-288, Konica Minolta, Tokyo, Japan) and a spectrum resolution of 10 nm. On the CIE color scale, L* indicates the degree of lightness or darkness from perfect black (0) to almost perfect white (100); a* indicates the degree of redness (+) and greenness (−); and b* indicates the degree of yellowness (+) and blueness (−). Calibration of the spectrometer was carried out with a standard white plate of L*a* b*.

### 2.3. Growing of Japonica Rice and the Environmental Impact

The climatic factors important for *Japonica* rice cultivation reported in previous studies [45,46,47,48,49] were compared among two countries with the highest global cultivation of this variety (Republic of Korea and Japan) and the three countries with the highest levels of malnutrition globally (India, Bangladesh, and Nigeria). The compared factors were temperature, sunshine, and precipitation. Additionally, data on the impact of cultivating the two major varieties of rice (*Indica* and *Japonica*) on the environment were retrieved from published studies, analyzed, and discussed.

### 2.4. Statistical Analyses

**Figure A1:**
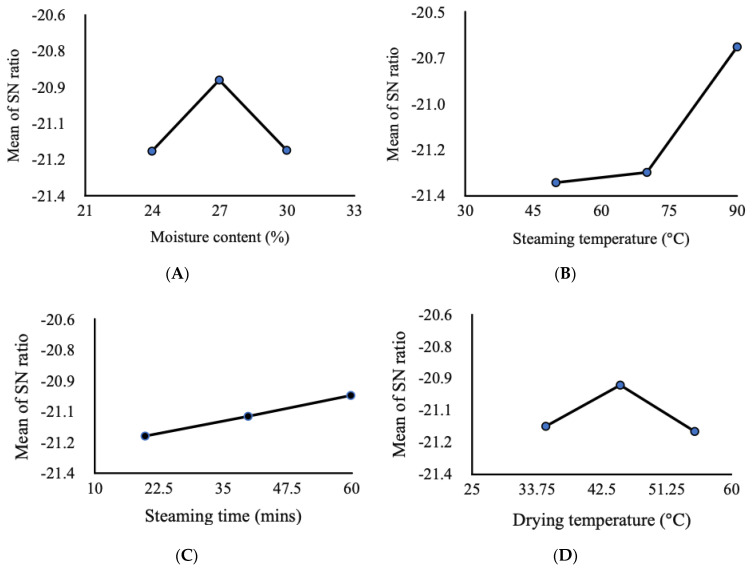
Main effects plot (data means) for SN ratios showing factors with the most impact on adhesiveness of *Japonica* rice: (**A**) moisture content, (**B**) steaming temperature, (**C**) steaming time, (**D**) drying temperature.

The impacts of moisture content after soaking, steaming temperature, steaming time, and drying temperature on the adhesiveness of *Japonica* rice were analyzed using the Taguchi orthogonal array in Minitab^®^ Version 14. Using this method, the factors affecting adhesiveness were ranked from 1 to 4, with 1 having the most significant effect and 4, the least, and represented by graphs (Figure A1). Further categorizations were carried out using the signal to noise (S/N) ratio in Equations (5)–(7). The smaller the S/N ratio in Equation (5), the more significant the impact, while the converse was true for Equation (7). For Equation (6), a nominal value was taken as the best indication of a strong impact on adhesiveness.

S/N = −10 × log(Σ(Y^2^)/n) (5)

where Y is responses for the given factor level combination, and n, the number of responses in the factor level combination.

S/N = −10 × log(s^2^) (6)

where s is the standard deviation of the responses for all noise factors for the given factor level combination.

S/N = −10 × log(Σ(1/Y^2^)/n) (7)

where responses for the given factor level combination and number of responses in the factor level combination were Y and n, respectively.

The significant differences (*p* ≤ 0.05) in measured nutrients among non-parboiled and parboiled *Japonica* and parboiled *Indica* rice were assessed using single-factor analysis of variance. Where significant differences were found, mean comparisons were carried out post hoc with Tukey’s honestly significant difference test. The nutrients were protein, fat, carbohydrates, iron (Fe), magnesium (Mg), and zinc (Zn). Ash content was also compared among the rice samples.

## 3. Results

### 3.1. Rice Attributes Preferred by Consumers

**Figure 3:**
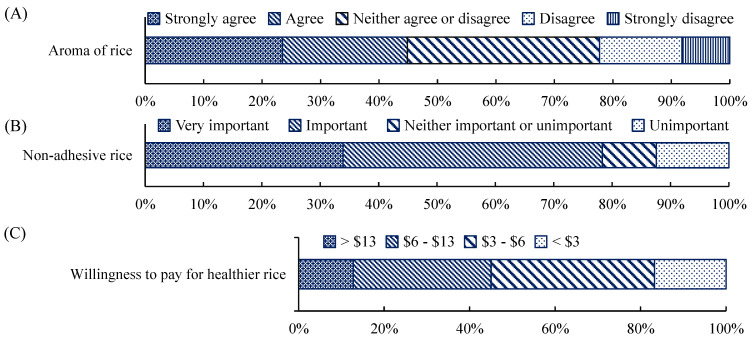
Survey respondents’ preference for rice based on (**A**) aroma, (**B**) adhesiveness, and (**C**) costs in Nigeria (West Africa) per 50 kg of rice (USD 1 = NGN 410).

Survey respondents (*n* = 1057) lived across the six geopolitical regions of Nigeria and ranged in age from 16 to 61 years, with 44.3% being female and 55.7% male. Their occupations included farming, manufacturing, and service provision. Sixty-eight percent of those surveyed were aware of the different rice varieties, 28% were not, and the remaining 4% were unsure. Respondents showed a varied preference for the rice properties tested; 32.9% were indifferent, 44.9% showed a preference for aroma, and 23.5% strongly preferred rice with aroma (Figure 3A). Generally, a total of 74.4% indicated that the non-adhesive rice was an important preference, with 32.2% indicating that it strongly was (Figure 3B). About 80% of respondents were also willing to pay a moderately higher price for non-adhesive rice (Figure 3C).

### 3.2. Effects of Parboiling on the Physiochemical Properties of Rice Samples

*a*.  *Adhesiveness and amylose content of milled parboiled and non-parboiled rice samples*

After one hour of the beakers of cooked rice samples standing upside-down on a flat surface, the contents of the beakers with the parboiled *Japonica* and *Indica* rice samples dropped onto the surface (Figure 2). In contrast, the non-parboiled rice sample remained at the bottom of the beaker. Parboiling reduced the adhesiveness of *Japonica* rice and increased the amylose content at all levels of treatment, as shown in Table 3. The Taguchi orthogonal array results showed that steaming temperature and moisture content after soaking were the factors with the highest impact on the adhesiveness of the parboiled *Japonica* rice (Figure A1).

*b*.  *Alkali digestion in milled parboiled and non-parboiled rice samples*

**Figure 4:**
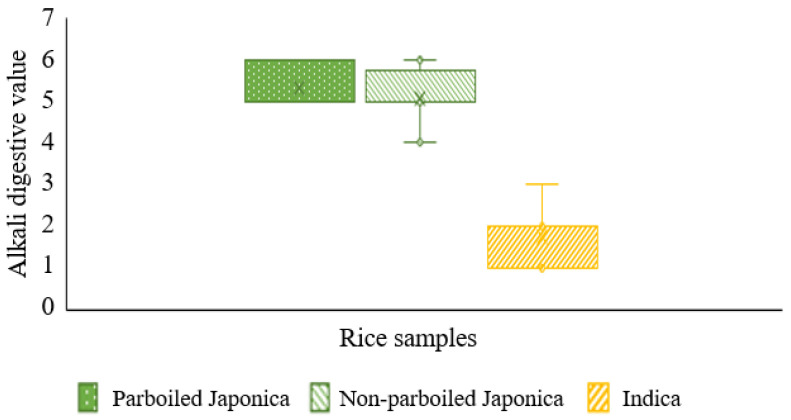
Kruskal–Wallis test for alkali digestion value of parboiled and non-parboiled *Japonica* rice and *Indica* rice.

The sample medians were 2, 5, and 5 for *Indica* (*n* = 12), parboiled *Japonica* (*n* = 12), and non-parboiled *Japonica* (*n* = 12), respectively (Figure 4). Both *p*-values (adjusted and non-adjusted for ties) observed were less than the significance level of 0.05, indicating that the median quality index differed in at least one of the *Indica* samples, and post hoc tests confirmed this.

*c*.  *Nutrient levels observed in milled parboiled and non-parboiled rice samples*

**Figure 5:**
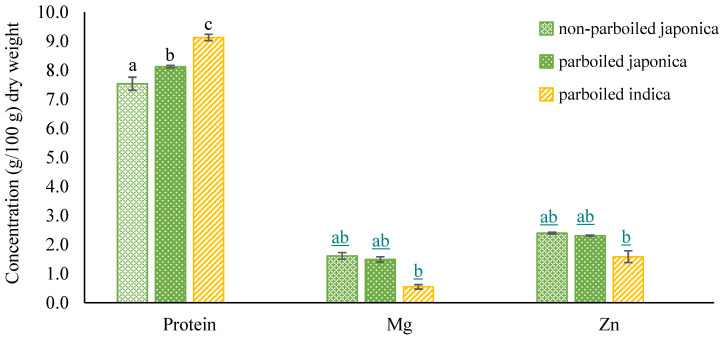
Bar graphs showing mean protein and micronutrient (Mg and Zn, mg/100 g) concentrations in non-parboiled *Japonica* (*n* = 3), parboiled *Japonica* (*n* = 3), and *Indica* (*n* = 3) rice samples. Error bars show standard deviations. Mean concentrations with different letters are significantly different (*p* ≤ 0.05).

The carbohydrate, fat, and ash contents measured in rice samples were comparable in non-parboiled *Japonica*, parboiled *Japonica*, and parboiled *Indica* (Table 4). The concentration of the micronutrient Fe was also similar in the measured rice samples (Table 4). Protein was highest in the parboiled *Indica* samples and lowest in the non-parboiled *Japonica* (*p* ≤ 0.05; Figure 5) at concentrations of 9.1 ± 0.03 g/100 g and 7.5 ± 0.03 g/100 g, respectively. However, magnesium and Zn levels were lowest in the parboiled *Indica* samples while similar in both *Japonica* rice samples (*p* ≤ 0.05; Figure 5). Heinemann et al. [50] reported protein concentrations of 6.85 ± 0.34% and 6.76 ± 0.20% in non-parboiled and parboiled brown rice, respectively, with a moisture content of 12.6 ± 0.54% for non-parboiled and 12.07 ± 0.7% parboiled brown rice.

*d*.  *Color of milled parboiled and non-parboiled rice samples*

**Figure 6:**
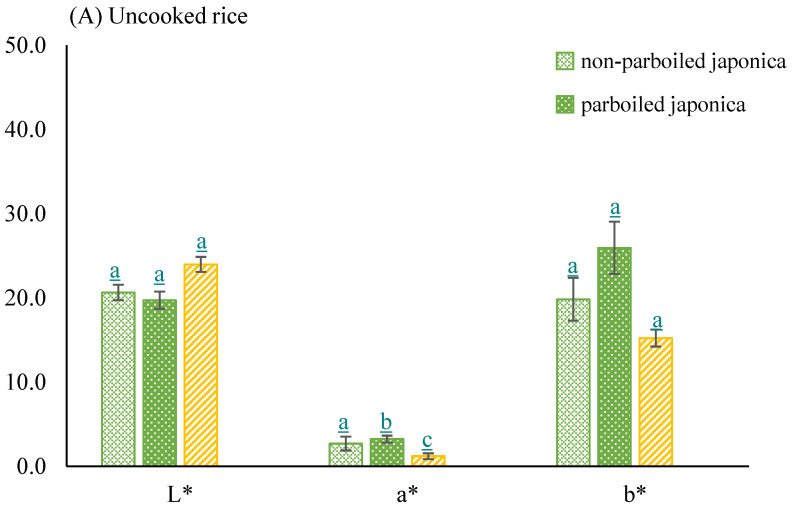

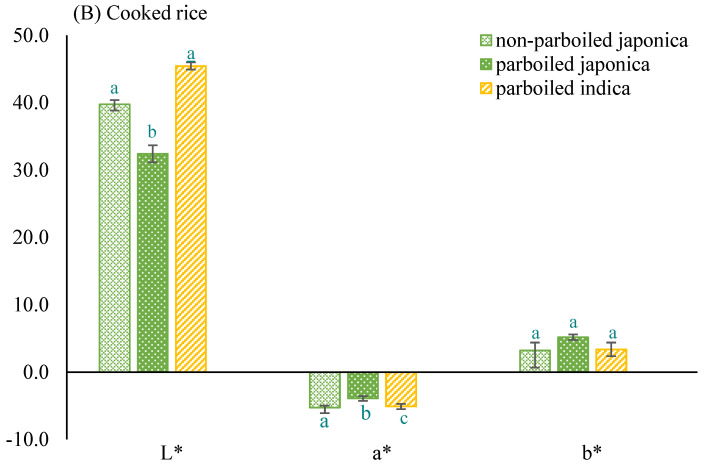
Bar graphs showing mean CIE L*a* b* in (**A**) cooked and (**B**) uncooked non-parboiled *Japonica* (*n* = 5), parboiled *Japonica* (*n* = 5), and *Indica* (*n* = 5) rice samples. Error bars show standard deviations. Means with different letters are significantly different (*p* ≤ 0.05).

The degrees of whiteness (L) and yellowness (b) were similar among all three uncooked rice sample types. Whiteness values ranged between 18.8 in parboiled *Japonica* and 24.2 in *Indica* rice (Figure 6A). The degree of redness was significantly different (*p* ≤ 0.05) in all three uncooked rice sample types with average values of 1.2 ± 0.35, 2.7 ± 0.82, and 3.22 ± 0.42 for parboiled *Indica*, non-parboiled *Japonica*, and parboiled *Japonica*, respectively. The whiteness increased when samples were cooked with *Indica*, still scoring the highest at 45.5 ± 0.54, although the differences among all cooked rice types were not statistically significant, *p* ≤ 0.05 (Figure 6B). Redness changed into greenness in all three samples when cooked, but the differences remained significant (*p* ≤ 0.05). Redness values recorded were −5.10 ± 0.39, 5.26 ± 0.29, and −3.92 ± 0.34 for parboiled *Indica*, non-parboiled *Japonica*, and parboiled *Japonica*, respectively.

### 3.3. Growing Conditions of Japonica Rice in Different Rice-Consuming Regions

**Figure 7:**
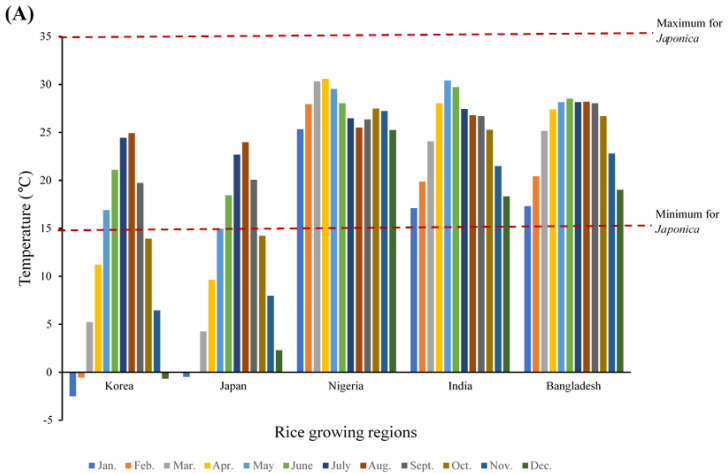

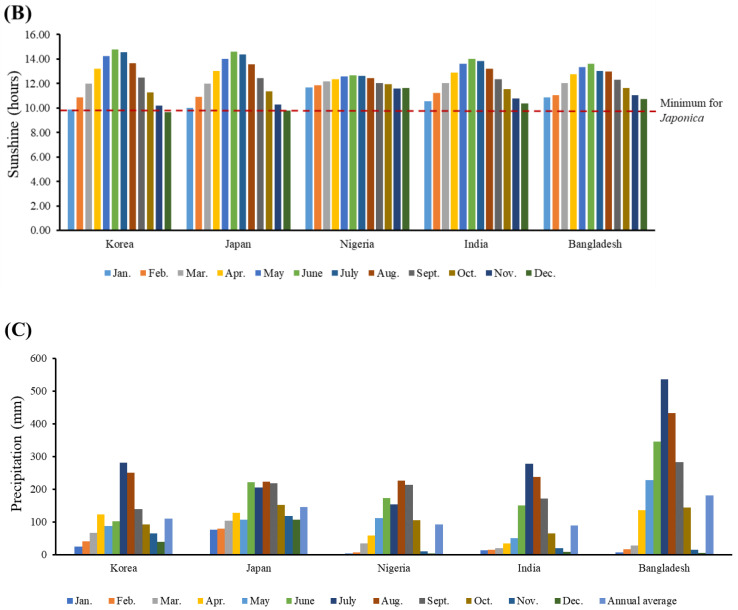
Major factors for growing *Japonica* rice: (**A**) mean temperature, (**B**) sunshine, and (**C**) precipitation [45,46].

The climatic factors important for *Japonica* rice cultivation (temperature, sunshine, and precipitation) which have been reported in previous studies [45,46]. for five countries are presented in Figure 7. The Republic of Korea and Japan are the two countries with the highest global cultivation of *Japonica* rice. India, Bangladesh, and Nigeria are the three countries with the highest levels of malnutrition globally. Published data on the greenhouse gas impacts of cultivating the two major varieties of rice (*Indica* and *Japonica*) were also reviewed and the implications for food-insecure regions are discussed below.

## 4. Discussion

### 4.1. Rice Quality Attributes Preferred by Surveyed Consumers and the Effects of Parboiling on Physicochemical Properties

A larger proportion of the *Indica* rice-consuming population surveyed preferred non-stickiness (non-adhesiveness) and the pleasant aroma of parboiled rice. The survey also showed that the reduced level of adhesiveness and aroma that comes with parboiled rice [50] formed important criteria in determining eating quality. The majority (about 80%) of respondents were willing to pay more for rice that was seen as non-adhesive. The main reasons given by survey respondents for their choices included ease in cooking, taste satisfaction, customs and traditions, and appearance after cooking.

As the parboiling treatment has been demonstrated to alter the physicochemical properties of *Japonica* rice [50] and optimize rice quality parameters [51,52], the impacts of the parboiling treatment on preferred characteristics were further investigated in *Japonica* rice.

Amylose content in rice correlates with its adhesiveness and is seen as a very important rice consumption parameter. The *Japonica* rice variety usually contains low amylose, which is associated with increased adhesiveness. Parboiling increased the amylose content at all level of treatment in this study. Chinnaswamy and Hanna [53] recorded amylose content of 16.1% for non-parboiled *Japonica* rice (control) and 23.1% for *Indica* rice. The same study saw elevated rice amylose levels with increasing processing temperature.

Adequate steaming and soaking of rice before parboiling has been shown to eliminate the breakage of rice kernels caused by internal cracks acquired during maturity that are invisible to the naked eye. Soaking enables the void spaces in the hull to be filled with water [54], causing water absorption and subsequent swelling of the starch granules. Furthermore, rapid and uniform water absorption is enabled by soaking [55] and can be controlled with temperature and duration of soaking to achieve the desired moisture content. Similar to soaking, the results of steaming are also dependent on the temperature of the steam and its duration but, in addition, pressure plays a role in the final quality of the steamed paddy [56]. Steaming has also been recorded to greatly impact the final color of the milled rice and the head rice (whole grain) yield because it helps in hardening the rice kernel after soaking [57]. The rice quality from steaming is dependent on the prior soaking process [58]. Improper hydration of the paddy prior to steaming results in a steep moisture gradient from the outer to the inner core layers of the paddy, leading to cracks rather than hardening of the grain [59]. These cracks will result in a substantial loss of head rice. This implies that the two important factors affecting the adhesiveness of the rice also have substantial impacts on the head rice recovery rate.

The average ranks in a Kruskal–Wallis test showed that *Indica* rice differed the most from the average rank for all observations of alkali digestion and was lower than the overall median. Alkali digestion was inferred to be an influencer of the gelatinization temperature of rice [60].

With the exception of Mg, the chemical components measured in this study were comparable to values reported for other rice varieties [50,51]. In addition to pretreatment, protein and micronutrient concentrations in rice may be attributed to differences in genetic makeup, soil-water nutrient availability, and agronomic conditions during cultivation and harvesting. Conflicting results have been reported by studies of linkages between protein and micronutrient concentrations in rice. In a study of the micronutrient content of 274 milled rice genotypes, Jiang et al. [61] reported significant linkages between rice protein content and the micronutrients Mg and Zn, whereas Yang et al. [62] found no associations in a study of 285 polished rice grain genotypes. In the current study, the Mg and Zn contents were both higher in the *Japonica* rice samples (Figure 5). This indicated that *Japonica* rice could be an important source of micronutrients in helping solve hidden hunger.

In terms of visual appeal, rice color is an important measure, as reported in this study’s survey and supported by [63]. White rice was preferred among consumers. The degree of whiteness increased when samples were cooked. Although *Indica* scored the highest, the differences among all cooked rice types were not statistically significant.

### 4.2. Growing Conditions Conducive for Japonica Rice in Different Rice-Consuming Regions

Since the domestication of Asian rice, it has been cultivated under diverse agroecological systems to meet different human demands [64,65]. This has resulted in a wide genetic diversity in rice around the world, as shown by molecular tools such as the analysis of restriction fragment length polymorphisms [66], simple sequence repeats [67], and genome-wide single nucleotide polymorphisms [68]. Consequently, many rice varieties with different characteristics have arisen under natural and human selection [69]. Although *Japonica* and *Indica* sub-species have been reported to have the same origin [70,71], they have been domesticated under widely different environmental conditions. Rice has evolved into different types, including upland and lowland paddy rice [72], as well as glutinous and non-glutinous rice [73]. Among these, *Indica* and *Japonica* rice represent the most important differentiated sub-species. For example, *Japonica* cultivars are mainly cultivated in temperate environments at high latitudes or altitudes with cool climatic conditions. In contrast, *Indica* cultivars are usually grown in tropical and sub-tropical regions at low latitudes and altitudes [74].

*Indica* and *Japonica* represent two partially isolated gene pools that serve as extremely valuable genetic resources in the improvement of rice varieties [75]. Being adapted to different environments, *Indica* and *Japonica* varieties have developed diverse morphological, agronomical, physiological, and molecular characteristics [76] that provide valuable genetic resources for breeding high-yielding rice [76]. Efficient utilization of such resources relies on understanding the genetic differentiation and geographical distribution patterns.

In [77], the relationships between average temperatures (range between 14 °C and 33 °C) at 17 rice cultivating sites with available precipitation and sunlight data and their latitudes (between 2° S to 44.5° N) were analyzed with a general linear model. This was carried out during the rice planting season (April–October) over 30 years (1961–1990). Furthermore, to estimate the relationships between the distribution of *Indica*, *Japonica*, and intermediate rice varieties and temperature, the average temperature correlation from nine latitude ranges against the percentage of alleles for every type of rice was analyzed. Results from [76] observed that as the latitude increased, the average temperature during the rice-growing season decreased. The study went on to examine the linkages between temperature and the spatial distribution of the different types of rice (i.e., *Indica*, *Japonica*, and intermediate) and found that, up to a point, with increasing temperature (and decreasing latitude), the proportion of *Indica* rice (in terms of total area cultivated) increased. This increase peaked at around 26 °C (corresponding to 30° N) and thereafter decreased with a further increase in latitudinal temperature, indicating that the distribution of *Indica* rice was not consistently correlated with the change in temperature. In contrast, *Japonica* rice varieties from different latitude ranges and their corresponding temperatures showed a negative correlation. This result indicated that *Japonica* rice, usually grown in temperate regions, is highly sensitive to temperature changes in terms of its geographical distribution across different latitudes.

*Japonica* rice is prevalently grown under sub-tropical and temperate climatic conditions, but in recent years, new strains have been adapted to tropical conditions and produced in countries such as Thailand and Vietnam. This could imply that the market segmentation into *Japonica* and *Indica* rice may become less marked over the longer term, with a larger number of countries able to shift from *Indica* to *Japonica* rice varieties or vice versa, as prices change.

Temperature is a major factor for rice cultivation, with its different growth stages varying in requirements. These include temperature for germination as follows: six days at 25 °C and two days at 27 °C to 37 °C. Lower temperatures take longer and the seedlings will not grow when it exceeds 40 °C [77]. At 30–35 °C, the plant height increases, the rate of grain growth is faster, and a shorter grain-filling period occurs [78,79]. In [80], low temperatures were reported to have a significant negative effect on all growth stages of rice of different varieties. In [80], three temperature classes (maximum, minimum, and optimum) for growing *Japonica* rice varieties were determined using leaf elongation rate. The base temperature (minimum) reported was between 13 °C and 14 °C, the maximum was between 38 °C and 42 °C, and the optimum was between 28 °C and 31 °C in most varieties.

*Japonica* rice is usually cultivated between May and September in the Republic of Korea and Japan. For five years (2012 to 2016), the temperatures required for rice plant development at different growth stages in the major *Japonica* rice-cultivating countries were recorded [80]. The Republic of Korea had a mean maximum temperature of 24.9 °C in August and the lowest of 16.9 °C in May (Figure 7A). As May is usually the first month of cultivation, this indicated that the farmers started the planting season in mild temperatures, which is suitable for germination but not necessarily the optimal, as stated above. A similar trend was recorded in Japan, with minimum and maximum mean temperatures ranging from 15.0 °C to 24.0 °C in May and August, respectively (Figure 7A). To achieve better performance, rice cultivation in Korea and Japan generally begins in nurseries with controlled temperatures and seedlings transplanted into fields as the weather warms during the planting season. The high energy costs and associated greenhouse gas contributions would be expected to be higher than in the tropical regions studied where controlled environments are not needed.

The amount of daily sunshine hours in the Republic of South Korea was a minimum of 13.7 h in August and 14.8 h in June, with a similar trend in Japan (Figure 7B). The Republic of South Korea and Japan had average annual precipitation of 109.2 mm and 144.8, respectively, as shown in Figure 7C.

In examining the temperatures in Africa (Figure 7A), the highest mean temperature in Nigeria for a period of five years (2012–2016) was 30.6 °C, and the lowest was 25.5 °C. This fell within the optimal temperature range required for *Japonica* rice [80]. The lowest and highest mean temperatures between 2012 and 2016 for Bangladesh and India were 17.3 °C and 28.5 °C, and 17.1 °C and 29.7 °C, respectively (Figure 7A). These data show a favorable climate for growing *Japonica* rice year-round in these countries with the world’s largest populations of resource-poor and malnourished people.

Furthermore, *Japonica* rice could be cultivated at least twice a year in a four-month cultivation cycle in tropical countries. In [81], it was reported that more sunlight would improve the productivity of the rice. Rice is recorded to need 9–12 h of light daily [81], but no specific amounts have been given for *Japonica* rice to the authors’ knowledge. Bangladesh, India, and Nigeria had the required amount of daily sunlight for rice growth all year round, as shown in Figure 7B. The only constraint in cultivating *Japonica* rice year-round in the high rice-consuming countries with vulnerable people might be the availability of water (Figure 7C). This problem could be mitigated by constructing dams and storing water during the peak raining periods for irrigation.

### 4.3. The Potential Contribution of Japonica Varieties to Greenhouse Gas Reduction

Agriculture had an estimated emission of 5.1 to 6.1 carbon dioxide equivalents per year (Gt CO_2_-eq/yr) in 2005. This is 10–12% of the global GHG total. Furthermore, about 60% and 50% of N_2_O and CH_4_, respectively, of global anthropogenic emissions came from agriculture [82]. Growing rice contributes 25–300 Tg/year, amounting to 10–20% of global methane emissions [83]. In [84,85], South East Asia, where *Indica* rice is massively cultivated, emitted about 10,000 kg of methane per square kilometer, contributing about 90% of the global total methane emissions from rice cultivation. Methane, a potent greenhouse gas, is produced in enormous quantities by bacteria in waterlogged rice fields through anaerobic decomposition of organic material. Nitrous oxide, a powerful greenhouse gas, is also produced by soil microbes in rice fields, but its impact on global warming has been given lesser attention than CH_4_ emissions from rice fields. The intensity of N_2_O emissions is related to nitrogen (N) fertilizer application rates [86].

Worldwide measurements show that there are large temporal variations in CH_4_ fluxes, and these differ distinctly with soil type and texture and the application of organic matter and mineral fertilizer [87]. The reduction in CO_2_ with H_2_, fatty acids, or alcohols as hydrogen donors, and the transmethylation of acetic acid or methanol by methane-producing bacteria, are the major pathways of CH_4_ production in flooded soils. In paddy fields, the kinetics of the reduction processes are strongly affected by the composition and texture of soil and its content of inorganic electron acceptors [88].

There are three main processes of CH_4_ release into the atmosphere from rice fields: diffusion loss of CH_4_ across the water surface (least significant), as bubbles (ebullition) from paddy soils (important during land clearing and especially if the soil texture is not clayey), and CH_4_ transport through rice plants. Plant transport of methane is the most critical loss mechanism, with more than 90% of total CH_4_ emitted during the cropping season being released by diffusive or lacunae transport through the aerenchyma system of the rice plants [89,90]. Rice plants develop an intercellular gas space system, the aerenchyma, which provides roots submerged in inundated soils with oxygen (O_2_). This gas space system also enables the transport of other gases, including CH_4_, N_2_O, and carbon dioxide (CO_2_), from the soil/sediment to the atmosphere. Consequently, the variation among rice varieties with different growth and developmental progress could result in differences in total GHG emissions among rice varieties [91].

The increasing demand for rice in the future has raised concerns about increasing GHG emissions [92,93]. Considering the importance of rice as a staple food crop which provides more calories to the global population than any other single crop, the cultivation of more climate-friendly varieties would help mitigate the impacts of greenhouse warming that might be associated with increased production.

In [89], substantial differences were observed in the impacts of rice variety type on greenhouse gas emissions, rice yield, and global warming potential (GWP). The CH_4_ and N_2_O emissions were 6356 and 267 kg CO_2_e/ha for *Indica* rice varieties and 4845 and 295 kg CO_2_e/ha for *Japonica* rice varieties, respectively. A statistically significant higher yield-scaled GWP occurred in *Indica* rice varieties (1101.72 kg CO_2_e/ha) than *Japonica* rice varieties (711.38 kg CO_2_e/ha), indicating that the *Japonica* rice varieties released less GHGs with higher yields. The inverse relationship between rice yield increases and GHG emission reductions will aid cropping technique innovation for rice production in achieving higher yield while lowering emissions.

## 5. Conclusions

Parboiling retained the micronutrient profile of the treated *Japonica* rice, indicating that it could be a potential solution to hidden hunger in rice-consuming countries. Adhesiveness was also reduced in treated *Japonica* rice, thereby providing a quality that most survey respondents found to be desirable. The increasing human population is expected to increase rice demand worldwide. With higher yields, the cultivation of *Japonica* rice varieties will likely have lower GHG emissions compared to *Indica.* With *Japonica* rice being more affected by temperature than geography, parts of Africa, India, and Bangladesh would have conducive weather for growing it. The adoption of the more nutritious higher-yielding *Japonica* rice has the potential to help strengthen global food security and reduce hidden hunger, particularly in the world’s five most impoverished countries, while also reducing the GHG emissions of global rice cultivation.

## Figures and Tables

**Table 1:** Conditions for parboiling *Japonica* rice.

Exp. No.	Moisture Content after Soaking (% Wet Basis)	Steaming Temperature (°C)	Steaming Duration (min)	Drying Temperature (°C)
1	24	50	20	35
2	24	70	40	45
3	24	90	60	55
4	27	70	20	55
5	27	90	40	35
6	27	50	60	45
7	30	90	20	45
8	30	50	40	55
9	30	70	60	35

**Table 2:** Alkali digestion value seven-point scale.

Degree of Digestion	Alkali Digestion Classification	Alkali Digestion Value
Grain not affected	Low	1
Grain swollen	Low	2
Grain swollen, collar incomplete and narrow	Low	3
Grain swollen, collar complete and wide	Intermediate	4
Grain split or segmented, collar complete and wide	Intermediate	5
Grain dispersed, merging with collar	High	6
Grain completely dispersed and intermingled	High	7

**Table 3:** Amylose content of parboiled and milled *Japonica* rice.

Treatment	1	2	3	4	5	6	7	8	9
Amylose (%)	17.93	18.33	19.03	18.53	19.43	18.20	19.37	18.37	18.87
S/N Value	25.07	25.26	25.59	25.36	25.77	25.20	25.74	25.28	25.51

**Table 4:** Mean and standard deviation (SD) of chemical constituents measured on a dry weight basis in *Japonica* and *Indica* rice samples.

Constituent	Units	Non-Parboiled *Japonica* (*n* = 3)	Parboiled *Japonica* (*n* = 3)	Parboiled *Indica* (*n* = 3)	Reported Values in Literature (Range)
		Mean (SD)	Mean (SD)	Mean (SD)	
Crude Protein	g/100 g	7.5 (0.23)	8.1 (0.11)	9.1 (0.03)	5.71–6.71 ^a^; 7.21–8.53 ^b^
Crude Fat	g/100 g	0.4 (0.23)	0.4 (0.23)	0.5 (0.28)	0.31–0.47 ^a^
Ash	g/100 g	0.5 (0.24)	0.5 (0.26)	0.5 (0.19)	0.49–0.60 ^a^
Carbohydrates	g/100 g	91.6 (0.28)	90.9 (0.54)	89.9 (0.44)	
Fe	mg/100 g	0.8 (0.18)	0.9 (0.23)	1.0 (0.13)	0.12–0.78 ^a^
Mg	mg/100 g	1.6 (0.04)	1.5 (0.09)	0.5 (0.02)	
Zn	mg/100 g	2.4 (0.11)	2.3 (0.08)	1.6 (0.20)	0.84–1.46 ^a^

(^a^) Kwarteng et al. [51] and (^b^) Heinemann et al. [50].

## Data Availability

The data presented in this study are available in the article.

## Appendix B

- **(Survey Questionnaire)**

**Personal Data**

- Age:
- Gender:
- Occupation:
- Country of residence:
- State of residence:

**Survey questions**

1. Do you know there are varieties of rice? Yes/No/Not sure
  a. If yes, what type(s):
  b. Which of the above type do you prefer?

2. The length of rice is important to me.
  (a) Strongly Agree (b) Agree (c) Neither Agree nor Disagree (d) Disagree (e) Strongly Disagree

3. Why is the length of rice important to you?
4. Adhesiveness (stickiness) of rice is important to me.
  (a) Strongly Agree (b) Agree (c) Neither Agree nor Disagree (d) Disagree (e) Strongly Disagree

5. Why is the adhesiveness of rice important to you?
6. Why is the adhesiveness of rice not important to you?
7. The aroma of rice is important to me.
  (a) Very Important (b) Important (c) Neither Important nor Unimportant (d) Unimportant (e) Very Unimportant

8. The price of rice is important in your decision for selecting the brand of rice to buy.
  (a) Very Important (b) Important (c) Neither Important nor Unimportant (d) Unimportant (e) Very Unimportant

9. What do you look out for when selecting rice? (Multiple selections is applicable)
  (a) Rice brand (b) Length of the grain (c) Adhesiveness after cooking (d) Aroma (e) Price (f) Others (please list)

10. What is the maximum you could pay extra per 10kg bag of rice if you knew it was healthier?
  (a) Less than ₦1000 (b) ₦1000–2000 (c) ₦3000–4000 (d) More than ₦5000

11. Which source of rice do you prefer?
  (a) Foreign (b) Local (c) Any

12. Why do you prefer your option in question 11 above?
